# Ocean nomads or island specialists? Culturally driven habitat partitioning contrasts in scale between geographically isolated sperm whale populations

**DOI:** 10.1098/rsos.211737

**Published:** 2022-05-18

**Authors:** Felicia Vachon, Taylor A. Hersh, Luke Rendell, Shane Gero, Hal Whitehead

**Affiliations:** ^1^ Department of Biology, Dalhousie University, Halifax, Canada; ^2^ Max Planck Institute for Psycholinguistics, Nijmegen, The Netherlands; ^3^ Sea Mammal Research Unit, University of St Andrews, School of Biology, St Andrews, UK; ^4^ Department of Biology, Carleton University, Ottawa, Canada; ^5^ Department of Zoophysiology, Aarhus University, Aarhus, Denmark

**Keywords:** culture, population structure, sperm whale, cultural segregation, distribution, scale

## Abstract

The sperm whale (*Physeter macrocephalus*) is a deep-diving cetacean with a global distribution and a multi-leveled, culturally segregated, social structure. While sperm whales have previously been described as ‘ocean nomads’, this might not be universal. We conducted surveys of sperm whales along the Lesser Antilles to document the acoustic repertoires, movements and distributions of Eastern Caribbean (EC) sperm whale cultural groups (called vocal clans). In addition to documenting a potential third vocal clan in the EC, we found strong evidence of fine-scale habitat partitioning between vocal clans with scales of horizontal movements an order of magnitude smaller than from comparable studies on Eastern Tropical Pacific sperm whales. These results suggest that sperm whales can display cultural ecological specialization and habitat partitioning on flexible spatial scales according to local conditions and broadens our perception of the ecological flexibility of the species. This study highlights the importance of incorporating multiple temporal and spatial scales to understand the impact of culture on ecological adaptability, as well as the dangers of extrapolating results across geographical areas and cultural groups.

## 1. Introduction

Levin [1] highlighted that scale is a fundamental problem in ecology and that, to truly understand a system, we need to incorporate information from multiple scales. This is particularly relevant to the study of animal population structure as populations can be structured on multiple scales according to their genetics [2], life history [3], environment/distribution [4] and/or culture [5–7]. It is important to understand population structure, and the scale at which it occurs, from an ecological and conservation standpoint as individuals within a subdivided population might have different behaviours, habitat use and/or resource use, and, therefore, different exposure to human threats and conservation needs. Furthermore, the scale-dependent structural diversity of a species may reflect diversity in inherited information (cultural and genetic) that can be an important element of overall biodiversity. However, the large spatial and temporal scales over which many cetacean species live their lives create significant challenges to understanding their populations and ecology [8]. This is especially the case in species like the sperm whale (*Physeter macrocephalus*), which can travel thousands of kilometres [9,10] and has a complex, multi-level social structure [11].

Sperm whales are a deep-diving marine predator with a worldwide distribution [11,12]. Female sperm whales generally inhabit tropical pelagic waters while males disperse to high latitudes at the onset of maturity [11]. Females have a multi-level social structure centred around social units of one or two matrilines [13,14]. These social units usually contain 6–12 individuals and are stable over time [15–17]. Multiple social units will sometimes join each other for periods of hours to days, forming groups, which forage or socialize together [18,19]. Interactions between social units are strongly influenced by their membership of cultural groups, called vocal clans, which we distinguish based on their distinctive repertoires of coda vocalizations [20,21].

Codas are stereotyped patterns of clicks [22] used by sperm whales in social contexts [23]. Codas vary in their length (usually between 3 and 12 clicks), rhythmic pattern and tempo, and can be classified into types based on their inter-click intervals (ICIs) [24]. The coda type repertoire of a social unit is made up of all the coda types that the unit produces, while the coda usage repertoire refers to the relative contributions of those types to the overall vocal output (following methods of Hersh *et al*. [25]). These repertoires are stable over time [26–28] and can be used to identify vocal clans, thought to represent a higher order social structure, by differentiating between social units that share coda usage repertoires and those that do not [20]. Vocal clans have been documented worldwide: in the Eastern Tropical Pacific (ETP) [26], off the island of Dominica in the Caribbean [21], off Japan [29], off Brazil [30] and off Mauritius [31]. Whales from different vocal clans have distinct coda type and usage repertoires and generally do not associate with each other, even when they occur in sympatry. Variation in these dialects is not consistent with genetic variation [13,32], indicating that coda repertoires are socially learned. Vocal clans are therefore suggested to be a culturally mediated form of population structure [32,33].

In the Eastern Caribbean (EC), research has been conducted since 2005 in the waters leeward of the island of Dominica by the Dominica Sperm Whale Project (DSWP). During this time, the DSWP documented the social structure and behaviour of 19 known social units [16,34] and identified two vocal clans: EC1 and EC2 [21]. However, one vocal clan (EC1) made up 97% of the documented 937 photo-identified sperm whale encounters between 2005 and 2019, with the second vocal clan (EC2) encountered so rarely it was only recently recognized as a distinct clan (i.e. 2016), although records show it has been present in the region for at least as long as EC1 [21].

Around the Galápagos Islands, the presence of a rare vocal clan was a precursor to a large-scale population shift (the replacement of sympatric vocal clans Regular and Plus-One by sympatric vocal clans Short and Four-plus) [35], but there are no suggestions of such changes in the EC as the same EC1 social units are still regularly encountered by the DSWP [36]. The presence of a second, rare vocal clan off Dominica thus raised general questions about regional EC sperm whale clan structure—how prevalent is the EC2 clan?—and globally—over which scales do the processes generating vocal clans operate?

Here, we address these questions by expanding the spatial scale of the EC research effort in two ways: first, by extending research beyond Dominica to encompass most of the Lesser Antilles; and second, by comparing the spatial scales of movement and population structure of sperm whales in the EC to those in the ETP.

## 2. Methods

### 2.1. Field methods

The waters around the Lesser Antilles were surveyed from a 12 m auxiliary sailboat between the island of St. Kitts & Nevis and Grenada following three predefined transect lines: leeward inshore (5–7 nautical miles from coast), leeward offshore (15 nautical miles from coast) and windward inshore (5–7 nautical miles from coast) (electronic supplementary material, figure S1). A total of eight approximately two-week long surveys were conducted from St. Lucia between the months of February and April in 2019 and January and March in 2020.

During the surveys, which were carried out whenever possible under sail, we used a two-element hydrophone array (two high-frequency Magrec HPO3 elements with high-pass filter set at 2 kHz) towed behind the vessel on a 100 m cable to record sound continuously via a Fireface UC or UMC202HD USB audio interface connected to a PC computer running the PAMGuard software [37], sampling at 96 kHz (this allows for the detection of most whale and dolphin species and clear sperm whale recordings). We listened to the hydrophone through PAMGuard every 30 min to determine whether there were sperm whales or other cetaceans in the area. When echolocation clicks of sperm whales were heard, groups of sperm whales were followed acoustically (using the towed hydrophone with bearings estimated by the Click Detector module in PAMGuard and/or a mechanical directional hydrophone) and visually (during daylight hours) for periods of hours up to 1 day. Most, but not all, acoustic detections of sperm whales led to visual detection. Photographs of flukes were taken using DSLR cameras with 300 mm lenses for individual identification purposes [38], GPS fixes were obtained every 5 min through a GPS marine chart plotter (Standard Horizon in 2019 and Raymarine in 2020), and vocalizations were recorded continuously using the towed hydrophone. The boat travelled continuously (day and night) at an average speed of four knots. Whales were approached slowly from behind, no closer than 150 m (unless whales voluntarily approached the vessel), to limit disturbance. We spent more time with groups of females compared to single mature males, and with individuals for which we did not have prior photo-identification data (based on comparison with an offline DSWP photo-identification catalogue available on board: 240 individuals from 34 social units/groups identified between 2005 and 2018). If the sea conditions allowed, we stayed with unknown groups until we heard at least 80 codas, to ensure that we would be able to extract and analyse at least 25 codas [26] and had multiple repeat photographs of individuals' flukes.

Codas extracted from acoustic recordings from the 2019 and 2020 surveys were pooled with those from the DSWP (2005–2018) and the Watkins Marine Mammal Sound Database [39], which were recorded between 1981 and 1995 off Dominica, Canouan, Bequia, and St. Lucia. Together, they comprise a sample of EC sperm whale codas covering a significant temporal (1981–2020) and spatial (St. Kitts & Nevis to Grenada, approximately 600 km) scale.

### 2.2. Delineating groups and social units

A quality rating (Q) from 1 to 5 was given to each sperm whale fluke photograph [38,40] and only photographs with Q ≥ 3 were included in this analysis. All flukes (even those that were initially assigned in the field) were matched against known Caribbean individuals using the software Flukebook [41] (https://www.flukebook.org/). Newly identified individuals were considered part of the same group (i.e. a short-term joining of at least two social units, lasting a few hours to a few days) if they were identified on the same day and had coordinated general behaviour and movement [16]. As such, newly encountered sperm whales are assumed to be part of a group until we can confirm that the membership of all individuals is stable, at which point they are considered part of the same social unit. The criteria used to distinguish social units from groups has varied over time [16,18] but we use the most restrictive definition here: members of a group which have been documented associating (i.e. observed within 2 h of each other) in at least two different years are then defined as a social unit [16]. This definition is the same as that used by the DSWP [16] and is more stringent than that used in the ETP, which considers groups social units if they are re-identified over a timescale of at least 30 days [18]. In this paper, we describe both social units and groups as not all newly encountered groups of sperm whales met the further requirements of stable membership in the definition of a social unit.

### 2.3. Vocal clan membership

Recordings were analysed by trained auditors and codas were manually marked using CodaSorter (K. Beedholm, Aarhus University), a custom written LabView (National Instruments, TX, USA) program implemented in MATLAB. Only repertoires of 25 or more codas and only codas with 3–11 clicks were included in the analysis, following previous methods [26]. Three- to 11-click codas constituted the bulk (98.9%) of marked codas and only considering them accounts for the potential of inconsistent marking of very short (less than three clicks) or very long (greater than or equal to 12 clicks) codas. All codas recorded on the same day were pooled together to form a repertoire that was assigned to the group of sperm whales identified on that day. Codas from days with multiple encountered social units were considered single repertoires and assigned to the combination of social units [26].

To delineate vocal clans across social units and groups, we compared coda repertoires using the identity call method, IDcall [25] to identify characteristic coda types, termed ‘identity codas’ (i.e. coda types that are used consistently by one set of repertoires and rarely by others). Codas were first separated according to their number of clicks and then classified into types according to their absolute ICIs using parsimonious mixtures of multi-variate contaminated normal distributions (R package ‘ContaminatedMixt’) [42]. Vocal clans were then delineated based on differences in the presence and usage of identity coda types in repertoires [25]. We tested various combinations of the parameters of the IDcall method to show that our identification of vocal clans was robust to parameter variation (electronic supplementary material, table S1).

### 2.4. Movement analyses

We used the track of our research vessel as we followed groups of female sperm whales during the 2019 and 2020 surveys to measure fine-scale EC sperm whale movements. Tracks were broken into 1 h, 3 h and 6 h segments to compare movement across vocal clans. Displacement was measured as the shortest distance between two GPS fixes (5 min resolution). To account for any bias caused by the research vessel moving purposefully away from a group of whales while still in acoustic contact, only the portion of the vessel track which occurred between the time of the first and last sightings of sperm whale clusters (animals at the surface within 30 m of each other and displaying coordinated behaviour) [11] on any day were included in this analysis. For longer timescales (days, months and years), we used the likelihood method from Whitehead [43] to describe patterns of movement. Photo-identification data collected during the 2019 and 2020 surveys were used to estimate root-mean-squared (RMS) displacement (an estimate of the shortest distance covered by an individual across a specific time lag based on its locations) across multiple time lags. The likelihood method corrects for an uneven distribution of effort and is appropriate for this analysis given the spatial variability in our survey effort. Only time lags greater than one day were included in this analysis since sperm whales were actively tracked for up to one day and, therefore, photo-identifications within a day are autocorrelated [43]. Error bars for RMS displacements were obtained using individual jackknifing, whereby individual sperm whales were removed from the analysis in turn and RMS displacement was recalculated [44]. We only included adult female and immature sperm whales in this analysis because calves were not reliably identified in the field, and mature males have very low re-sighting rates in the Caribbean [16,19,21] and display very different movement patterns [45]. This analysis was carried out using the continuous movement module of the SOCPROG software [46].

### 2.5. Comparison with Eastern Tropical Pacific sperm whales

Finally, we compared our findings with data from previous studies describing the social structure (i.e. mean social unit size, mean typical group size) [17,47], movements (i.e. hourly to yearly displacement, range) [9,43,48] and distribution (i.e. vocal clans' range and potential overlap) [26,49] of ETP sperm whales. These data were collected in the ETP between 1985 and 2014 using very similar protocols (i.e. dedicated, vessel-based acoustic and visual sperm whale surveys), and analysed using similar methods (e.g. group and social unit definition, vocal clan assignation, RMS displacement), making our comparisons valid.

## 3. Results

Over the 2019 and 2020 surveys, we obtained sperm whale photo-identifications on 56 days (24 in 2019; 32 in 2020), during which we obtained 778 h of sperm whale recordings (339 in 2019; 439 in 2020) and 13 394 photographs (5197 in 2019; 8197 in 2020). From 4 267 photographs with a quality score of Q ≥ 3, we photo-identified 214 adult sperm whales, 145 of which were not in existing EC catalogues (DSWP and other Flukebook contributors). These newly identified whales were from 23 groups (averaging six photo-identified adult individuals each), of which four qualified as social units using our definition. Our photo-identification results suggest that we encountered individuals that rarely, if ever, use the waters off Dominica, since expanding our research effort considerably increased the total number of identified individuals (from 536 whales identified over 15 years by the DSWP to 681, electronic supplementary material, figure S2).

### 3.1. Acoustic data

From the 2019 and 2020 survey recordings, we marked 5558 codas from 31 groups (23 newly discovered, and 8 which had previously been documented by the DSWP). These data were pooled with 13 years of acoustic data recorded primarily off Dominica from the DSWP (11 375 codas from 19 well-known social units), and data from the Watkins database (2106 codas from 21 days between 1981 and 1995) giving a combined dataset of 19 039 codas. Of these, 163 codas were excluded from the analysis because they contained more than 11 clicks and 813 codas were excluded because they were from usage repertoires with fewer than 25 codas. The final dataset thus had 18 063 codas comprising 151 different usage repertoires that we used to identify vocal clans (electronic supplementary material, table S2).

**Figure 1.**
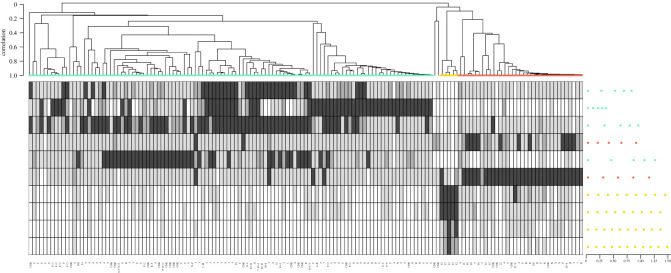
Average linkage hierarchical clustering dendrogram (top) depicting acoustic similarity among the three EC sperm whale vocal clans: EC1 (blue), EC2 (red) and EC3 (yellow). Each branch corresponds to the coda repertoire of a certain group/social unit of sperm whales on a certain day (corresponding groups/social units labelled underneath). Each row of the heat map (bottom) shows probabilistic usage by repertoire of each identity coda type. Heat map shading corresponds to the percentage of the repertoire made up of each identity coda type with white 0%, light grey 0–5%, grey 5–10% and dark grey 10% or higher. Identity coda types are depicted to the right of the heat map by dots representing each click in the coda and are coloured according to clan with duration in seconds underneath. See electronic supplementary material, figure S3 for a version of this diagram showing all coda types.

The 42 groups (22 of which qualify as social units) of sperm whales included in this analysis (31 groups from the 2019 and 2020 surveys and 11 groups from DSWP data that were not encountered during the surveys) were divided into three different acoustic clades according to their use of 10 identified identity coda types (figure 1; electronic supplementary material, figure S3). Our analysis recovered the previously documented EC1 and EC2 vocal clans, with EC1 repertoires dominated by the 1 + 1 + 3 coda type (54% of the recorded EC1 codas) and EC2 repertoires containing predominantly 5R and 2 + 1 + 1 + 1 codas (combining to 61% of the recorded EC2 codas). However, while EC2 was previously rarely encountered off Dominica (2.5% of all DSWP encounters between 2005 and 2019, electronic supplementary material, table S3) and therefore assumed to be uncommon in the EC [21], our results suggest that, on a regional scale, they are as numerous as EC1 groups—10 groups of EC1 whales and 11 groups of EC2 whales were identified during the 2019 and 2020 surveys and, cumulatively (including DSWP data), 18 distinct groups of EC1 whales and 14 distinct groups of EC2 whales have been identified in the EC (most of which qualify as social units).

Furthermore, we found evidence for a potential third vocal clan (EC3) represented by a single social unit (Unit 12; comprised 10 adults and 2 calves) with a coda usage repertoire dominated by long, regular identity codas, with 9R, 10R and 11R types making up 57% of the recorded EC3 repertoire.

### 3.2. Distribution and movements

Over our 2019 and 2020 surveys, we had a total of 50 encounters with sperm whales that lasted from 4.5 h to 28 h (mean 14 h). Twenty-four of these encounters were with EC1, 22 with EC2, 5 with EC3 and 1 with both EC2 and EC3. We had more encounters than groups (50 encounters with 31 groups) as some groups were encountered on multiple days. In total, we had acoustic track data that spanned 771 h, making up the 9249 GPS fixes that were used to map the kernel density of EC vocal clans throughout the Lesser Antilles.

**Figure 2.**
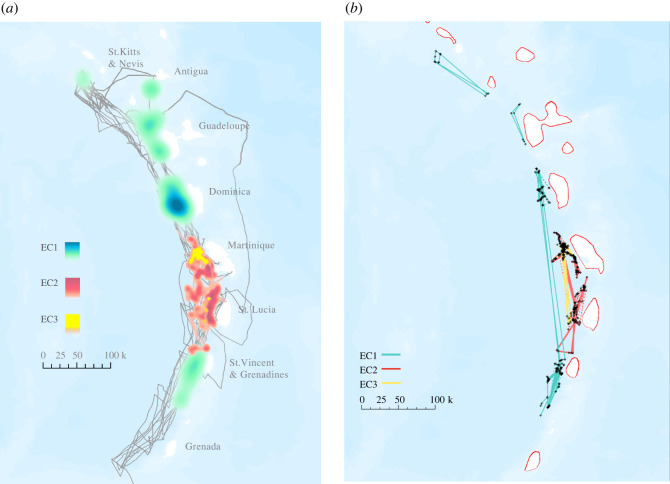
(*a*) Kernel density distribution of EC vocal clans with track of the research vessel (light grey). We calculated the density of acoustic encounters with groups/social units from each clan at a 0.001° resolution (approximately 100 m) using the kernel density spatial tool in ArcGIS. (*b*) Movement of photo-identified sperm whales between 2019 and 2020. Each dot corresponds to an individual identification. Full lines represent movement across years while dotted lines represent movement within years.

In the study area, very consistent winds (the ‘trade winds’) divide the marine habitat around the Lesser Antilles into windward (east) and leeward (west) zones. All our sperm whale encounters were to leeward of the islands, with higher encounter rates around the central islands of Martinique, Dominica and St. Lucia. There was a clear divide in the distribution of the vocal clans, with little overlap between EC1 whales (encountered around St. Kitts & Nevis, Antigua, Guadeloupe, Dominica, and St Vincent & the Grenadines) and EC2 whales (encountered around Martinique and St Lucia). The EC3 unit's distribution overlapped with EC2, being mostly sighted off Martinique but also encountered off St. Lucia (figure 2).

EC sperm whales' mean and maximum displacement over 1 h, 3 h and 6 h was similar across vocal clans, with about 3–4 km displacement per hour (electronic supplementary material, figure S4). Estimates of movement over longer timescales from photo-identification data suggest that RMS displacement increased over time lags of up to one year, with daily displacements in the range of 10 to 20 km (figure 4). EC1 whales had slightly higher displacement then EC2 whales using the likelihood methods from Whitehead [43] (electronic supplementary material, figure S5) and tended to be re-sighted more consistently around the same island, although a few made larger movements along the Lesser Antilles (figure 2). For instance, 74% (17/23) of the EC1 individuals re-sighted across survey years were re-sighted off the same island, while this number decreases to 41.7% (5/12) for EC2. These data suggest that, overall, sperm whales in the EC have a high degree of residency over our two-year survey timescale, with most displacements ranging across only one or two islands between years. This is consistent with the high re-sighting rate of EC1 social units in Dominica by the DSWP [36], the increase in the number of known individuals resulting from expanding our research area to include additional islands, and the fact that only one of the 26 known DSWP EC1 social units (Unit J) was encountered outside Dominica and Guadeloupe waters during our surveys.

### 3.3. Comparison with Eastern Tropical Pacific

**Figure 3.**
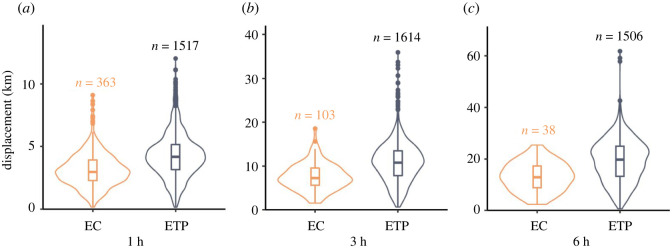
Violin plots displaying the (*a*) 1 h, (*b*) 3 h and (*c*) 6 h displacement of sperm whales from the EC and ETP. Sample size is displayed above each violin plot.

**Figure 4.**
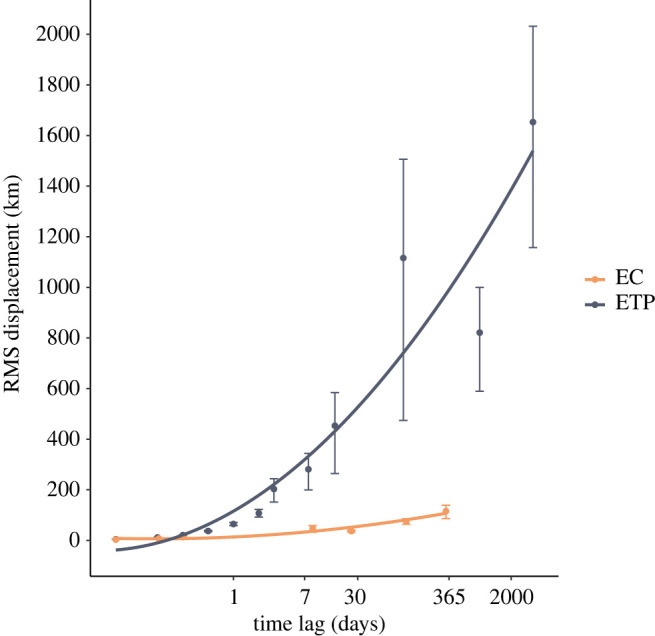
RMS displacement of EC sperm whales (orange) and ETP sperm whales (dark grey) over increasing time lags. Error bars display jackknife standard error. RMS displacement was calculated using the likelihood method [43] for time lags beyond one day for EC data and two days for ETP data.

Across all time scales, EC sperm whales had lower displacement than ETP sperm whales. This became more apparent as the time lag increased, from 1 h (mean 36.1% lower), to 3 h (mean 42.7% lower) to 6 h (mean 45.9% lower) (figure 3) and as we included daily and yearly displacement through photo-identification. Over timescales of up to a year, the residency of EC sperm whales contrasts starkly with the movement of ETP sperm whales. The difference in RMS displacement between the two geographical regions increases dramatically over time scales of a few hours to years to reach a factor of 10 difference over timescales of one year (100 km versus 1000 km mean displacement; figure 4).

A similar trend can be observed at the vocal clan level, with the EC1 and EC2 vocal clans having lower mean and maximum 1 h, 3 h and 6 h displacements (electronic supplementary material, figure S4) and lower RMS displacement across all time lags (electronic supplementary material, figure S5) compared to ETP vocal clans.

## 4. Discussion

### 4.1. Third vocal clan: a new vocal clan?

We are cautious in our description of EC3 as a new vocal clan, given that it is comprised of a single social unit (Unit 12). This social unit was encountered on six different days over two years, one day of which was with Unit Y, an EC2 social unit. This is particularly unusual as encounters with two vocal clans (EC1 and EC2) on the same day have only been reported four times over 15 years by the DSWP (0.004% of photo-identification encounters). However, while Unit 12 (EC3) and Unit Y (EC2) individuals were photographed within 16 min of each other on February 17th 2020, only one whale from Unit 12 (IDN 6432) was observed in cluster (measure of association: when whales are within 30 m of each other and moving in a coordinated fashion [16]) with individuals from Unit Y (IDN 6223 and IDN 6221). Throughout that day, even if both groups were in the same area, individuals chose to disproportionally associate (in clusters) with individuals from their own vocal clan: out of the 18 clusters observed, only one contained individuals from both vocal clans. Therefore, it is possible that EC2 and EC3 ranges overlap, but that whales choose to disproportionally associate with individuals from their own vocal clan. Alternatively, EC3 could be actively branching off from EC2 or be an EC2 social unit with a highly stereotypical social unit coda repertoire [28]. As more evidence accumulates, so will our certainty of the existence of EC3 as a distinct sperm whale vocal clan. However, the fact that the coda repertoire and membership of the EC3 social unit were stable across years, and the former distinctive from both the EC1 and EC2 repertoires (with long, fast, regular identity codas and almost no overlap with identity codas of EC1 and EC2; figure 1) suggests that a third vocal clan could be using the study area.

### 4.2. Ocean nomads?

Sperm whales have been described as ‘ocean nomads’, with male and female movements on the order of a thousand kilometres recorded in the ETP [9,50], North Pacific [10] and Mediterranean [51]. Our results show, however, that sperm whales in the EC organize their societies on a much smaller geographic scale. EC vocal clans have distinctive distributions around the Lesser Antilles over spatial scales of up to a few hundreds of kilometres and temporal scales of at least two years (figure 2). Social units tend to stay around the same one or two islands with high re-sighting rates within-islands and rare between-islands movements of up to 270 km (figure 2). Dominica, which we assumed was a good representation of the EC sperm whale population, therefore only represents a biased subset. This is surprising as the spatial scales of individual movements, social unit movements, and the spatial distribution of clans are on the order of thousands of kilometres in the ETP [9,26,48], and the Lesser Antilles span around 600 kilometres.

We also confirmed previous work that showed that EC social units and group sizes are relatively small compared to the ETP, with a mean social unit size of 6.8 individuals for EC1 [16], 8.1 individuals for EC2 (this study), and group sizes in the range of 7–9 individuals, compared with mean social unit sizes of 10–14 individuals and mean group sizes of about 30 individuals for the ETP [15,16] (table 1). This adds to accumulating evidence of the variable social structure of sperm whales on a global scale, with differences not only between the Pacific and Atlantic [15], but also the Mediterranean [51] and Mauritius (Indian Ocean) [14].

**Table 1.** Summary of principal differences in scale between sperm whale vocal clans of the EC (EC1, EC2) and ETP (Regular, Plus-One). Presented as mean ± s.d. when appropriate.

	EC	ETP
number of vocal clans	2–3	5^a^
number of individuals in vocal clans	hundreds	on the order of 10 000^a^
mean social unit size	EC1: 6.8 ± 2.8 (3–12)^b^	Regular: 13.6 ± 7.0^c^
	EC2: 8.1 ± 2.5 (6–13)	Plus-One: 10.7 ± 4.2^c^
mean typical group size	7–9^b^	30.4^d^
3 h displacement	EC1: 8.0 ± 0.9 km	Regular: 10.2 ± 2.4 km^e^
	EC2: 7.0 ± 0.8 km	Plus-One: 10.7 ± 0.2 km^e^
6 h displacement	EC1: 13.7 ± 2.6 km	Regular: 16.8 ± 4.6 km^e^
	EC2: 12.3 ± 2.4 km	Plus-One: 19.4 ± 0.6 km^e^
daily RMS displacement	10–20 km	50 km^e,f^
maximum displacement of female	300–400 km	5000 km^e^
range	few hundreds of kilometres	1000–2000 km^e,f^

^a^[26].

^b^[16].

^c^[17].

^d^[47].

^e^[9].

^f^[43].

The vocal clan distribution and movement results from our 2019 and 2020 surveys are remarkable, with no overlap between EC1 and EC2 vocal clan distributions and very few long-range movements between years (figure 2). These results are corroborated by 15 years of DSWP data, which documented high residency of predominantly EC1 social units of Dominica [36] and never identified the 145 individual sperm whales that occupy neighbouring islands. However, over longer timescales, the lack of physical barriers in the ocean means such sharp delineations are unlikely to be impermeable. For instance, EC2 groups have been encountered, although rarely, outside of the waters of St Lucia and Martinique by the DSWP in the past (electronic supplementary material, table S3, 2.6% of total DSWP encounters). Similarly, female movements on the order of hundreds of kilometres, while extremely rare, have been documented (e.g. Dominica to St Lucia—35, Dominica to St Vincent—this study; Bahamas to Azores, Gulf of Mexico to Bahama—52). This highlights a caveat in this study: results presented here identify regional scale spatial patterns over a relatively small timescale. Nonetheless our results suggest that there is an approximate order of magnitude difference in the scales of movements and range spans of EC and ETP sperm whales, with much larger groups of whales covering much greater areas in the ETP compared to the EC (table 1). EC sperm whales display a level of fidelity in their habitat choice and a degree of fine-scale habitat partitioning between cultural groups previously undocumented for females of this species, changing our perspective on how stereotypical sperm whale ecology and movement is worldwide. Such differences in scale between the ETP and the EC could be driven by cultural differences in movement patterns between vocal clans (e.g. [4]) and/or some type of response to differences in the distribution and ecology of sperm whales' main prey (squids) between the two areas. Sperm whales in the ETP have been documented to prey on Humboldt squid (*Dosidicus gigas*) [52,53]—a highly mobile, migratory, species [54] which might be more broadly distributed than squid species in the EC. While studies of sperm whale diet are lacking in the EC, both neritic and pelagic squid species have been identified in the area (e.g. *Ommastrephes bartramii* and *Thysanoteuthis rhombus* [55,56]) and a reliance on potentially patchier [55] neritic species could explain variation in the scale of sperm whale movement across the two geographical areas.

However, the observations that EC vocal clans were substantially restricted, during our two-year survey period, to specific islands (or pairs of neighbouring islands) in the Lesser Antilles, and that these preferences are maintained in sympatry without evidence of nuclear genetic differentiation [13], suggest that differences in habitat use between EC1 and EC2 are mostly culturally driven. The exact mechanism responsible for such a divide remains unknown and is an important focus for future research, but we consider several potential explanations for this spatio-temporal pattern of behavioural variation below.

#### 4.2.1. Territoriality

Vocal clans occupy different territories which they defend from each other.

While territoriality is a widespread phenomenon in terrestrial mammals and can result in observable differences in distributions [57,58], this explanation seems unlikely for sperm whales, as the three-dimensional structure and seasonal, patchy resources of marine environments make them almost impossible to defend [59]. Furthermore, there has been no record of aggression between female sperm whales, which would be expected if vocal clans defended their territories from each other. Evidence of overlap between EC1 and EC2 ranges, such as sightings of EC2 units in Dominica and Guadeloupe by the DSWP (electronic supplementary material, table S3), also refutes this hypothesis.

#### 4.2.2. Prey-type specialization

Vocal clans have learned to use, and specialized on, prey which are distributed differently around the islands.

Prey-type specialization has been reported in several marine mammal species including killer whales (*Orcinus orca*) [60], sea otters (*Enhydra lutris*) [61] and bottlenose dolphins (*Tursiops* spp.) [62]. In the case of sperm whales, social learning abilities might have resulted in improved plasticity [63,64], or the spread of innovation [65] when encountering new prey types. Over time, this could lead to vocal clans exploiting different niches [7] with conformism reinforcing the divide in prey-type use [5]. This hypothesis makes ecological sense since other sperm whale traits, such as coda production, are thought to be maintained culturally over generational timescales [32]. Ecological specialization would theoretically decrease competition between vocal clans, and sperm whales have been shown to adapt to other ecological opportunities, such as the spread of fishery interactions [66]. However, prey-type specialization would only result in the distribution differences documented in this study if the prey on which the vocal clans are specialized are distributed differently across the different islands (for example, if Martinique and St. Lucia have different prey types than Dominica, Guadeloupe and St. Vincent), which is possible, but unlikely, given the ocean's general homogeneity over short spatial and temporal scales [67] and lack of evidence for island-specific species diversity in the Lesser Antilles [68].

#### 4.2.3. Geographic or habitat specialization

Vocal clans have accumulated knowledge on specific habitat types or areas and have learned to use them efficiently.

Similar to resource specialization, geographic or habitat specialization could occur if, instead of improving their use of a specific prey, different clans preferentially selected particular areas (i.e. geographic specialization), or the features characteristic of particular areas, such as bathymetry or current flow (i.e. habitat specialization). For geographic or habitat specialization to be ecologically beneficial, it would require the presence of predictable resources that are tied to the geography of an area or to particular habitats. This might be the case in the Lesser Antilles, which are characterized by predictable winds [69], a fairly consistent inflow from the Atlantic to the Caribbean Sea through the channels between islands [70], and a bathymetry that can vary dramatically from one island to the next. The more predictable resources and more heterogeneous bathymetry of the EC compared to the ETP may have led to the higher residency of sperm whales in particular areas. Such fine-scale habitat choice is, perhaps, more akin to the predictable use of certain higher latitude canyons by male sperm whales [71–73].

We suggest that geographic and/or habitat specialization, transmitted through social learning within units and clans (i.e. culture), is the most parsimonious explanation for the fine-scale ecology of EC sperm whales. Geographic and/or habitat specialization is most likely learned socially within social units within vocal clans, with certain social units showing high residency to certain islands and vocal clans having distinctive distributions in the Lesser Antilles as a whole. This is similar to African elephants (*Loxodonta africana*), which also display socially learned patterns of site and resource use [74]. Geographic or habitat specialization could account for differences in the scale of movements between EC sperm whales and ETP sperm whales, but also the general isolation of East and West Mediterranean sperm whales [75] and their fine-scale, bathymetry-related distribution [76]. This demonstrates more ecological diversity in the sperm whale species as a whole than was previously assumed.

Our suggestion of cultural geographic or habitat specialization implies that EC sperm whales use much smaller areas of habitat than sperm whales in the Pacific. This has three important implications for conservation. First, since current population estimates of the EC sperm whale population are based primarily on sightings off Dominica, they might not accurately reflect the regional situation. The threats identified for the Dominica sperm whales, and resulting in EC1 population decline [77], most likely apply to sperm whales with residency around different islands but need to be more thoroughly examined and assessed. Second, it raises the potential importance of protecting sperm whale vocal clans independently off the different islands in order to maintain cultural diversity and population resilience [78]. Finally, it highlights the dangers of extrapolating data across geographical areas and cultural groups.

While this study is limited in its temporal and spatial scale, it does show that sperm whales, often characterized as having a relatively uniform ecology [79] compared to other cetacean species [80], may yet show considerable variability in how they use their environment, being adaptively successful both as ocean nomads and as local specialists. As more temporal and spatial scales of data are incorporated into the study of sperm whales, we may find more diverse ecological strategies and more ways in which culture shapes their lives.

## Data Availability

The data are provided in the electronic supplementary material [81].
